# Nitrogen Backbone Oligomers

**DOI:** 10.1038/srep13239

**Published:** 2015-08-19

**Authors:** Hongbo Wang, Mikhail I. Eremets, Ivan Troyan, Hanyu Liu, Yanming Ma, Luc Vereecken

**Affiliations:** 1 Max Planck Institute for Chemistry, Biogeochemistry Department, PO Box 3060, 55020 Mainz, Germany; 2 State Key Lab of Superhard Materials, Jilin University, Changchun 130012, P. R. China; 3 Institute of Crystallography, Russian Academy of Sciences, Leninsky pr. 59, Moscow 119333, Russia

## Abstract

We found that nitrogen and hydrogen directly react at room temperature and pressures of ~35 GPa forming chains of single-bonded nitrogen atom with the rest of the bonds terminated with hydrogen atoms - as identified by IR absorption, Raman, X-ray diffraction experiments and theoretical calculations. At releasing pressures below ~10 GPa, the product transforms into hydrazine. Our findings might open a way for the practical synthesis of these extremely high energetic materials as the formation of nitrogen-hydrogen compounds is favorable already at pressures above 2 GPa according to the calculations.

Nitrogen is a unique element in terms of its huge difference in binding energy between nitrogen atoms attached with single (or double) bonds, versus the very strong, short triple bond N≡N. This latter bond (−477 kJ mol^−1^) is one of the strongest chemical bonds known, while the N−N bond is much weaker (−80 kJ mol^−1^)^1^. Because of this, many nitrogen compounds are high energy density materials (HEDM) that release a large amount of energy at their, possibly explosive, decomposition to the most stable species – dinitrogen molecules^2^. All-nitrogen crystal, the so-called “polymeric nitrogen,” has been formed at very high pressures as a three-dimensional crystalline^1^,^3^ or as a disordered network of single-bonded nitrogen atoms^4^. Chains of nitrogen atoms were also predicted^5^,^6^,^7^. Polymeric nitrogen is considered a material that can store an ultimately large amount of chemical energy. Unfortunately, megabar pressures (100 GPa) are needed to synthesize all-nitrogen polymers. This precludes any practical applications which should be in the pressure range of ~1 GPa or ideally ~0.1 GPa − the range of synthesis of ammonia in the Haber-Bosch process.

Another route for nitrogen high energy density materials is formation of nitrogen-hydrogen compounds. However, the weak single and double bonds prevent easy formation of large molecules or polymers. Recent work has discovered molecules with a larger number of nitrogen atoms in a row: N5^8^, N8^9^, and N10^10^; however, these compounds are either ionic or stabilized by carbon. Larger metastable polynitrogen molecules were predicted^11^ but not found experimentally yet.

Application of very high pressures dramatically changes the chemistry of nitrogen, where single covalent bonding becomes preferable. In the present work we explored a synthesis of single-bonded nitrogen-hydrogen compounds under high pressure with an idea that addition of hydrogen might reduce the pressure needed for synthesis of new energetic materials, and make these more stable in comparison to the purely nitrogen polymers. Hydronitrogen polymer was predicted by recent evolutionary *ab initio* calculations^12^: at pressures >36 GPa NH chains were formed from the ammonium azide precursor. Experimentally these calculations seem not to be supported as ammonium azide is stable at pressures of at least ~55 GPa at room temperature^13^, probably even higher pressure is required for the polymerization. In principle, diimide (NH)_2_ in which nitrogen atoms are double bonded might serve as a monomer but it is very unstable and difficult to handle. Finally, hydrazine does not polymerize as we observed no qualitative changes in the IR and Raman spectra by increasing the pressure up to 60 GPa and subsequently releasing the pressure (Fig. S1).

Because of the apparent lack of nitrogen-hydrogen precursors we explored a direct reaction using molecular nitrogen and hydrogen as the starting materials. Only a few studies on hydrogen-nitrogen mixtures under pressure are available. Changes in the Raman spectrum shows that hydrogen incorporates into the nitrogen lattice and interacts with the neighboring nitrogen atoms^14^,^15^,^16^. An inclusion compound (N_2_)_12_D_2_ was observed from a 1:9 D_2_:N_2_ mixture^16^. Ciezak *et al.*^15^ studied a 1:2 H_2_:N_2_ mixture at room temperature and found an indication for chemical transformation as new bands in Raman spectra appeared, which were assigned to N-N bending and stretching. On the other hand, molecular nitrogen and hydrogen vibrons were observed even at the highest pressures of 85 GPa, such that no definite conclusion was drawn on the behavior of this mixture^15^. Recently, Spalding *et al.*^17^ reported an amorphous dinitrogen network containing ionized ammonia at 50 GPa and room-temperature in a 1:1.17 N_2_:H_2_ mixture. However, recent theoretical work on the N_2_/NH_3_ mixture^18^ revealed that the polymeric N_2_H can be stable above 33 GPa.

In this work, we study H_2_/N_2_ mixtures at high pressures experimentally and theoretically, aiming for evidence of the formation of nitrogen backbone chains, and identifying the compounds formed.

## Experimental Results

**Figure 1:**
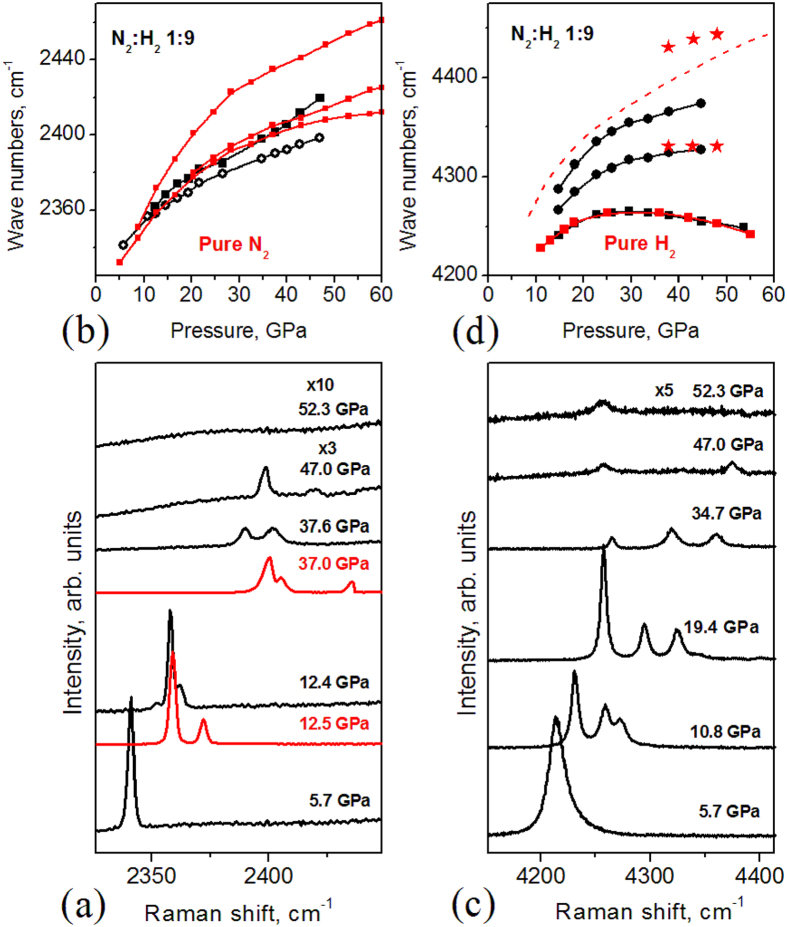
Nitrogen and hydrogen Raman spectra of N_2_:H_2_ mixtures at high pressures. (**a**) Raman vibron of nitrogen in the N_2_:H_2_ 1:9 mixture. The spectra are shifted vertically for better comparison. Note that Raman signals at pressures of 47 and 52.3 GPa are very weak and hence amplified three- and tenfold, respectively. Red lines are the spectra of pure nitrogen. (**b**) Pressure dependence of the Raman vibron of nitrogen in 1:9 N_2_:H_2_ mixtures compared to that in pure nitrogen (red points and lines). (**c**) Evolution of hydrogen vibrons with pressure in the 1:9 N_2_:H_2_ mixture. (**d**) Comparison of Raman vibrons of H_2_ in 1:9 N_2_:H_2_ mixture (black circles and squares) with IR^24^ (red dashed line) and Raman (red squares) vibrons of pure H_2_, and IR vibrons (red stars) of H_2_ in the 1:4 N_2_:H_2_ mixture.

We systematically studied N_2_/H_2_ mixtures with a wide range of nitrogen concentration ratios (5%, 10%, 20%, 50%, and 80%) as well as pure nitrogen and hydrogen at pressures of up to 70 GPa. For all mixtures we obtained consistent results and will illustrate results taken for different N_2_/H_2_ compositions. For instance, for the 1:9 N_2_/H_2_ mixture, the Raman spectra deviated from those of pure nitrogen and hydrogen at pressures as low as ~10 GPa: the nitrogen vibron peak split (Fig. 1a), and two strong satellites of the hydrogen vibron emerged (Fig. 1c) indicating that hydrogen and nitrogen are mixed and strongly interact with each other. At these pressures, however, they remained in the molecular state because vibron excitations in the Raman spectra persisted.

A major change occurs above ~35 GPa: the intensity of H_2_ and N_2_ Raman vibrons strongly decreases and then even disappears at ≅50 GPa (Fig. 1a–d, Figs S2, S3). This transformation is accompanied by a large decrease in volume, as indicated by the auto-decrease of pressure with time at high pressure (Fig. S3b). The new phase is amorphous because the X-ray diffraction pattern disappears at pressures above ~50 GPa (Fig. S2c,d).

**Figure 2:**
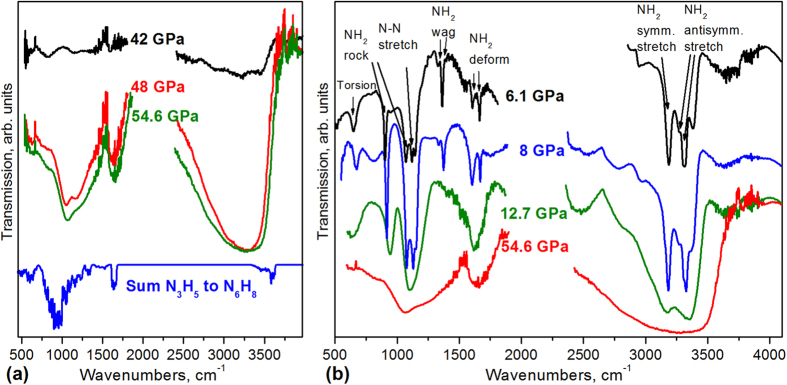
Infrared spectra of N_2_:H_2_ mixtures. (**a**) Infrared absorption spectra of N_2_:H_2_ 1:19 mixture with increase of pressure at 300 K. A broad absorption band around 3300 cm^−1^ appeared at 38 GPa (or 35 GPa in other runs with different mixtures). The oscillations in this spectrum are due to interference of light between parallel diamond anvil culets. The spectrum below (blue line) is the calculated sum absorption of oligomers from N3 to N6 chains in the gas phase. The peaks are sharp and narrow due to the absence of broadening interactions with the bulk material. Gas phase spectra of individual molecules as well as details of the calculations are presented in Figs S9–S16. (**b**) IR spectra of the 1:19 N_2_:H_2_ mixture at a releasing pressure after polymerization at 300 K. The spectra did not change qualitatively with pressure down to ~10 GPa. Below this pressure the spectra are identical to those of hydrazine (Fig. S4) and assigned according to Ref.^19^. Note that the comparison of spectra for the 1:4 and 1:1 N_2_:H_2_ mixture with the spectra of pure hydrazine is shown in Fig. S4.

**Figure 3:**
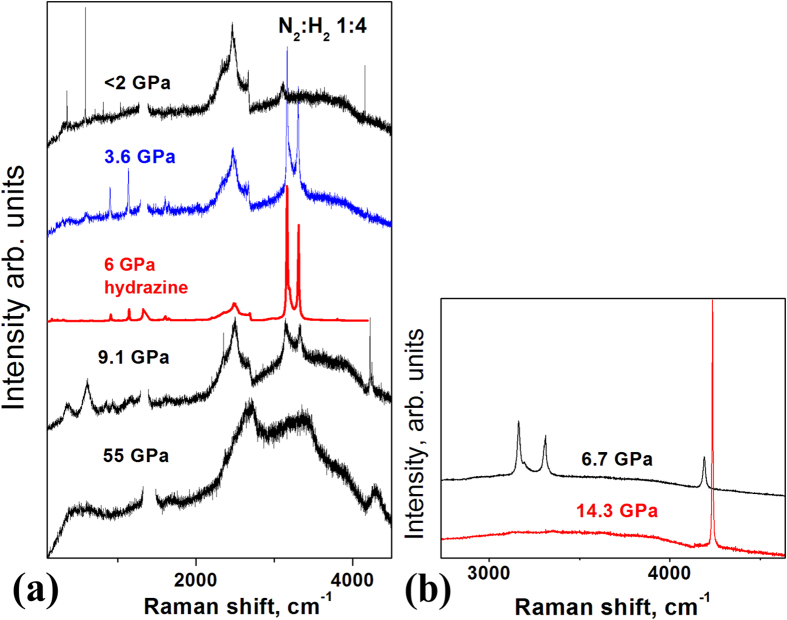
(**a**) Raman spectra of 1:4 N_2_:H_2_ mixture at the releasing pressure after reaction. At the highest pressures the Raman spectra reveal only broad bands around 3200 cm^−1^ and 4200 cm^−1^. At pressures below ~10 GPa sharp peaks developed from these bands, identified as hydrazine^19^ and hydrogen^24^. The increase in the intensity of the hydrazine peaks is accompanied by a decrease on the intensity of hydrogen vibrons as shown in detail in (**b**) for 1:19 N_2_:H_2_ mixture. Hydrazine decomposes producing hydrogen and ammonia by decreasing pressure below 2 GPa before opening the cell as seen in the sharp characteristic spectra.

IR spectroscopy provided us with several clues as to nature of the new phase. First of all, the strong absorption band at 3300 cm^−1^ (Fig. 2a) is characteristic of N−H vibrational stretching modes, indicating that the high-pressure transformation involves a chemical reaction between nitrogen and hydrogen. Second, upon releasing the pressure below ~10 GPa the product sharply transforms into hydrazine as it is unambiguously identified by the IR and Raman spectra (Figs 2b and 3)^19^. This transformation indicates that the product relates to hydrazine. However, the absence of torsion mode at ~600 cm^−1^ and wagging mode at ~1300 cm^−1^ (Fig. S4) indicates that longer molecules than hydrazine might be synthesized. However, precise identification of such a disordered chain-length material with spectroscopic and X-ray data alone cannot be done because the product is apparently is a disordered mixture of different molecules that results in the broad IR and Raman spectra (Figs 2 and 3) and the diffuse X-ray.

## Theoretical Calculations

To gain further insight in the possible nature of the high-pressure amorphous phase formed in the hydrogen-nitrogen mixture, we performed theoretical calculations based on two different approaches: metadynamics calculations^20^,^21^ to simulate structural transformations in the mixture and quantum chemical calculations to examine the energetics and spectrum of hydrogen-nitrogen molecules.

**Figure 4:**
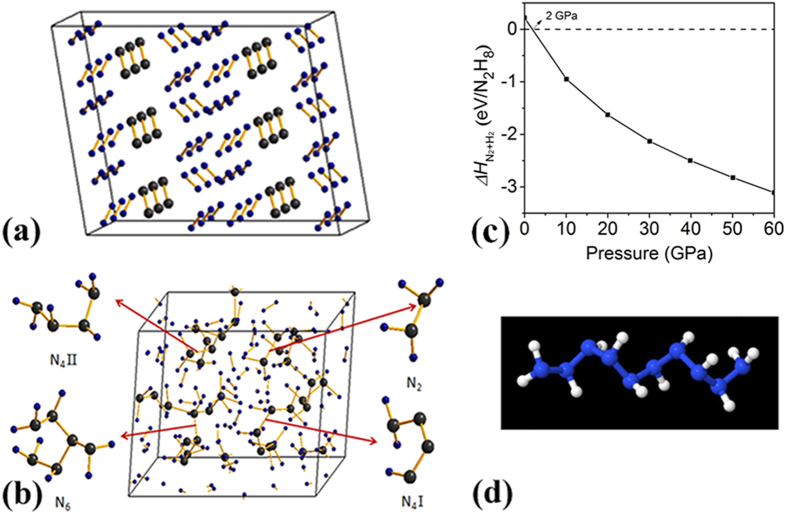
High pressure phase of the 1:4 N_2_:H_2_ mixture as predicted theoretically using the metadynamics method. The initial (**a**) and predicted (**b**) structures of N_36_H_144_ at 60 GPa and 300 K are displayed. Large and small spheres denote nitrogen and hydrogen atoms, respectively. (**c**) Calculated formation enthalpy of N-H compound with respect to the elemental decomposition into solid hydrogen (P6_3_/m) and nitrogen (R-3c) at 0 K and high pressures. (**d**) Ball and stick representation of N_10_H_12_, illustrating the low-energy spiral backbone structure.

Metadynamics simulations were performed at 300 K for a 1:4 ratio of N_2_:H_2_ in an initial mixed molecular arrangement (Fig. 4a). At 30 GPa no obvious structural changes or reactions were observed after 100 metasteps, which is typically long enough to model phase transitions, in good agreement with our experimental results. Upon compression to 60 GPa, reaction between nitrogen and hydrogen was found, where four kinds of hydronitrogen compounds were stabilized: H_2_N−NH_2_ (hydrazine), H_2_N−NH−NH−NH_2_ (N4-II), H_2_N−N=N−NH_2_ (labeled N4-I), and H_2_N−NH−N(NH_2_)−NH−NH_2_ (N6), in ratios of 7:3:1:1. Their formation is associated with a large enthalpy gain, about −298 kJ mol^−1^ per N_2_H_8_ formula unit (corresponding to the 1:4 N_2_:H_2_ mixture) relative to the reference N_2_ (*R*
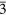
*c* structure) and H_2_ (*P*6_3_/*m*) (Fig. 4c). The formation of nitrogen-hydrogen compounds is favorable already at pressures above 2 GPa. However there is a large kinetic barrier for the polymerization while it is quite difficult to estimate its value. We have calculated the electronic band structure and density of states of N-H compounds at 60 GPa. The results showed that the formed N-H compounds are insulating with a band gap of ~3.7 eV (Fig. S5) rather than a metal predicted for a chain consisted only of nitrogen atoms^5^,^6^,^7^. Higher temperatures promote the polymerization: at 500 K, longer chains are created in larger amounts (Fig. S6).

Our quantum chemical calculations are based at the M06-2X/aug-cc-pVDZ level of theory (see Methods and the full description in the SI). We find that the azanes, the systematic class of N_n_H_n+2_ compounds analogous to the carbon-based alkanes, are energetically stable compounds even at zero pressure, in a qualitative agreement with the above *ab initio* calculations (Fig. 4). They have well-defined minima on the potential energy surface, and therefore can exist as separate compounds, even if their thermal lifetime is currently unknown. This also agrees with literature data^22^. Linear, unbranched polymeric chains, NH_2_−(NH)_n_−NH_2_, were studied for up to N10. Contrary to hydrocarbons which have a linear backbone, the most stable conformer of the azanes is a spiral-shaped nitrogen backbone (Fig. 4), owing to the interactions between the free electron pair and the bonds on the nitrogen atoms. Their formation enthalpy, relative to N_2_ and H_2_, increases by about 84 kJ mol^−1^ for every additional NH monomer, making azanes highly energetic materials with an energy density comparable to polymeric nitrogen.

The longer azanes were found to be thermally less stable than hydrazine. Thermal dissociation of hydrazine requires over 250 kJ mol^−1^, forming two ^•^NH_2_ radicals. For larger chains, >N4, breaking of the internal N-N bonds requires only 125 kJ mol^−1^, leading to the more stable −N^•^H radicals. This could indicate that longer azanes have a higher tendency to decompose to smaller compounds, even though the predicted barrier still affords long thermal lifetimes at 300 K. The formation, destruction and transformation of azanes appear to involve a complex set of radical reactions, which may be at work in the experimental setup. The full quantum chemical characterization of this chemistry is beyond the scope of this work.

## Discussions

Combining the experimental data with the theoretical predictions leads to further insights in the possible structure of the amorphous phase formed at high pressures. The metadynamics calculations show that NH-chains longer than hydrazine can be formed. The quantum chemical calculations on the formation enthalpies indicate that, per NH-unit, the formation enthalpies are very similar across longer azane chains. This suggests that, if hydrazine can be formed, there is no prohibitive energetic constraint in the formation of longer azanes. Upon release of pressure, the weaker internal nitrogen-nitrogen bonds are easier to break, allowing a rearrangement towards shorter chains, a process terminated by the formation of hydrazine that only incorporates a stronger terminal H_2_N−NH_2_ bond. This decomposition hypothesis is experimentally supported: first, upon pressure release starting from 50 GPa, the broad band in IR and Raman spectra are sharpening, which might indicate gradual transformation of longer chains to shorter chains. Furthermore, below 10 GPa hydrazine univocally dominates in the IR and Raman spectra (Figs 2b and 3, Fig. S4,S7). Second, the Raman H_2_ vibron intensity shows a decrease that accompanies the significant increase in the hydrazine lines in the Raman spectrum (Fig. 3b, Fig. S7).

We compare the theoretically predicted spectra for mixtures of azanes (Fig. 2, Figs S9-S16) against the experimental data (Fig. 2a, Fig. S4), finding interesting points of agreement. Firstly, the spectra predict a band of N−H stretch vibrations around 3500 cm^−1^. For hydrazine, this peak is well-structured, while in larger azanes, this peak broadens due to the wider variety of N-H moieties, where positioning in the chain, branching, and folding of the chains affect the individual N-H stretching modes. Compared to the higher-pressure spectra, the predicted peaks are not as broad, nor as intense, but this can be related to intermolecular interactions in the matrix. The same consideration is applied to a band around 1650 cm^−1^, calculated to be the NH_2_ deformation modes. This peak is found to be prominent in the spectra of all azanes, from N2 to N10, and broadens for more complex mixtures as a function of conformer folding, branching, and chain length. Finally, there are two broad peaks, 1500–700 cm^−1^, and below 700 cm^−1^, that match reasonably well with the observed spectrum. These modes are not easily assigned to specific motions as they often involve larger skeletal vibrations. Cyclic azanes, N_n_H_n_, were also examined. These, however, lack the distinct NH_2_ deformational peak around 1650 cm^−1^, and can thus be only a minor fraction of the products.

The formation of single-bonded hydrogen-nitrogen compounds was shown experimentally to occur at room temperature for pressures of about 35 GPa, opening novel ways to synthesize these high-energy materials. Compared to other high-energy materials, such as polymeric nitrogen, where polymer formation occurred at 150 GPa the needed pressures for synthesis are significantly lower. The pressure of polymerization might be further dramatically reduced as formation of the oligomer chains is energetically favorable at pressures as low as 2 GPa (Fig. 4c) according to the *ab initio* calculations and even at zero pressure according to the quantum chemical calculations. Increase of temperature is one of the ways to overcome the kinetic barrier of the reaction. Instead of pressure as was done at room temperature, we changed temperature at certain pressure. The transformation was monitored on the basis of the disappearance of the hydrogen vibron. Indeed, the pressure needed to induce formation of the new phase strongly reduces with temperature (Fig. S8) as we found by studying a 1:1 N_2_:H_2_ mixture. The IR absorption spectra were recorded after cooling to room temperature to verify the transformation. The pressure of the transformation drops with temperature approximately linearly. Owing to hydrogen diffusion out of the sample through the metallic gasket, we were unable to achieve higher temperatures. However a problem associated with high temperatures is formation of ammonia. Even at room temperature, we observed a small area of ammonia at the edge of the steel gasket (which is a catalyst) in few cases. The problem of separation of the N-H oligomers and ammonia and finding proper catalysts for the high temperatures remains to be solved. We believe that another method − ultraviolet illumination − might be effective for practical synthesis of the N-H oligomers at room temperatures and atmospheric or low pressures. UV radiation, for instance, from excimer laser with wavelength of 193 nm (~6.4 eV) can excite N_2_ and H_2_ molecules through one- or two-photon absorption to higher energetic states or break their bonds. Practical implementation of synthesis seems feasible with a complexity comparable to e.g. the Haber-Bosch process for synthesis of ammonia.

## Methods

We filled the diamond anvil cell with nitrogen/hydrogen mixtures with the aid of a gas loader at pressures of ~1500 bar. Typically we used gasket made of T301 steel. We checked if this material containing ~70% of Fe, 17% of Cr and 7% of Ni can act as a catalyst for the N_2_:H_2_ mixture. For that we used an insert made of NaCl and cleaned surface of diamonds from the rest of the gasket material. We obtained the same results as with the metallic gasket at room temperature.

We used type IIa synthetic diamond anvils for IR studies and low luminescence Ia diamonds for Raman studies. The Raman spectrometer was equipped with a nitrogen-cooled CCD, notch filters, and edge filters. The 632.8 nm line of a He-Ne laser, and the 647.1 nm and 676.4 nm lines of a krypton laser were used to excite the Raman spectra. Low-temperature measurements were performed in an optical cryostat. IR measurements were conducted using a Bruker IFS-66 V FTIR spectrometer equipped with a KBr beam splitter and a globar mid-infrared source. X-ray diffraction measurements were collected at the European Synchrotron Radiation Facility (ESRF, beamlines ID27) and Extreme Conditions Beamline at PETRA III, at DESY (Germany). The pressure was determined from the shift in the high-frequency edge of the Raman spectrum recorded from the stressed tip of the diamond anvil^23^ or with a ruby gauge^24^. The diamond anvil cell was heated with the aid of an external heater; the monitored pressure did not change appreciably.

Quantum chemical calculations were performed at the DFT level of theory, using the M06-2X functional^25^ in conjunction with the aug-cc-pVDZ basis set. This level of theory is expected to be sufficiently accurate to analyze the trends in properties across the chain length; sample higher level calculations for hydrazine were conducted but the differences were found to be not significant for our current purpose. All calculations were performed using the Gaussian-09 program suite.

In the present study, the metadynamics method was applied^20^,^21^ with the projector augmented plane-wave (PAW) method^26^, as implemented in the Vienna *ab initio* Simulation Package (VASP) code^27^. A PAW potential with a Perdew-Burke-Ernzerhof^28^ exchange-correlation functional was adopted. The simulation cells were constructed by using 18 nitrogen and 72 hydrogen molecules, and the Brillouin zone was sampled with Γ-point approximation. The canonical (*NVT*) ensemble was used for molecular dynamics runs. Each metastep of the metadynamics simulations comprised 600 time steps of 1.0 fs. Extensive metadynamics simulations with typically 100 metasteps for each simulation were conducted at pressures and temperatures of 30–60 GPa and 300–500 K, respectively. The width and height of the Gaussian bias potentials were *δ* = 30 (kbar Å^3^)^1/2^ and *W* = 900 kbar Å^3^, respectively. The metadynamics method^20^,^21^ is able to overcome barriers and hence can explore a broad range of candidate structures at finite temperatures. Successful applications of the method include several examples of reconstructive structural transitions^29^,^30^,^31^.

A plane wave energy cutoff of 600 eV was employed for the underlying *ab initio* structural relaxations. The k-point sampling of 4 × 3 × 4 for N_2_H_8_, 7 × 7 × 7 for N_2_ (*R*-3*c*) and 9 × 9 × 10 for H_2_ (*P*6_3_/*m*), respectively, were used to ensure that all the enthalpy calculations are well converged.

## Additional Information

**How to cite this article**: Wang, H. *et al.* Nitrogen Backbone Oligomers. *Sci. Rep.* **5**, 13239; doi: 10.1038/srep13239 (2015).

## Supplementary Material

Supplementary Information
