# Supplementary material for: Nitrogen Backbone Oligomers
Source: Sci Rep. 2015 Aug 19;5:13239. doi: 10.1038/srep13239 (PMC4541254; doi:10.1038/srep13239)
Supplement: Supplementary Information [file srep13239-s1.pdf]

Supplementary information

Nitrogen backbone oligomers

Hongbo Wang[1,2], Mikhail I. Eremets[1]*, Ivan Troyan[1,3], Hanyu Liu[2], Yanming Ma[2+], Luc Vereecken[1]

[1]*Max Planck Institute for Chemistry, Biogeochemistry Department, PO Box 3060, 55020 Mainz, Germany*

[2]*State Key Lab of Superhard Materials, Jilin University, Changchun 130012, P. R. China*

[3]*Institute of Crystallography, Russian Academy of Sciences, Leninsky pr. 59, Moscow 119333, Russia*

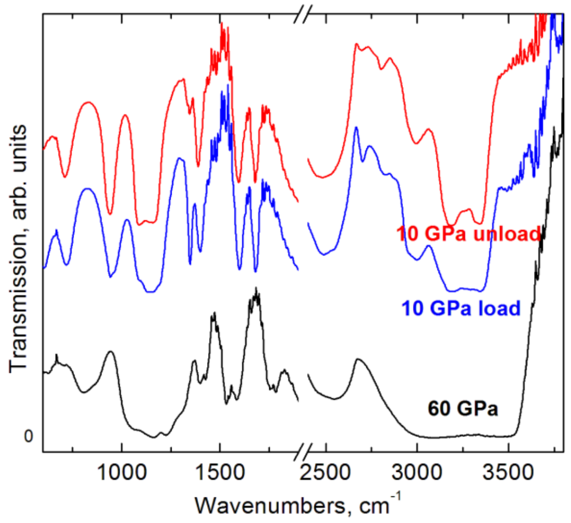

Fig. S1. Infrared spectra of hydrazine at selected pressures. The spectra gradually change with pressure and reversibly return back at releasing pressure. Spectra at intermediate pressures were omitted for clarity.

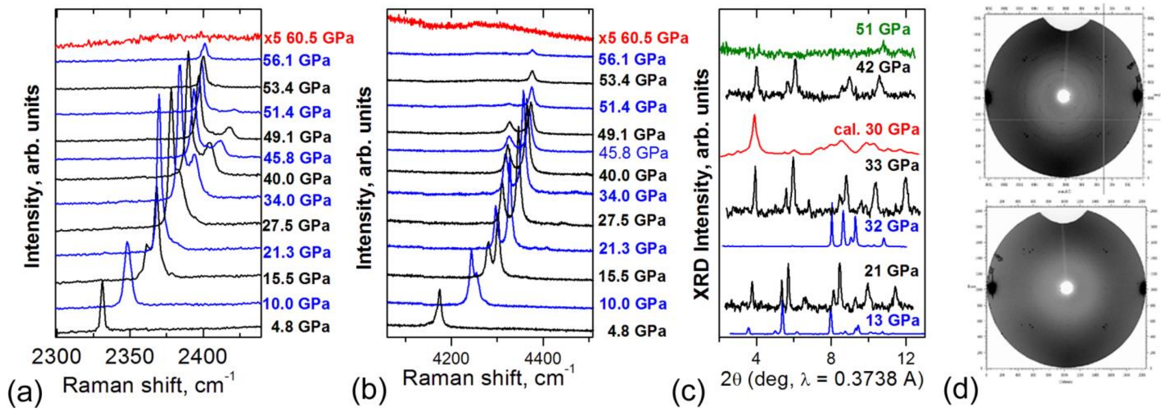

Fig. S2. Evolution of nitrogen (a) and hydrogen (b) vibron for the 1:4 N_2:H_2 mixture for increasing pressures. Note that the intensity of the 60.5 GPa spectrum was multiplied by five. (c) Experimental X-ray diffraction patterns of the 1:4 N_2:H_2 mixture (black lines) and pure nitrogen (blue lines) at different pressures. The diffraction pattern calculated from the predicted structure (see text) at 30 GPa is shown by the red curve. (d) Original images of power diffractions at 42 GPa (above) and 51 GPa (below) taken with oscillations E10°.

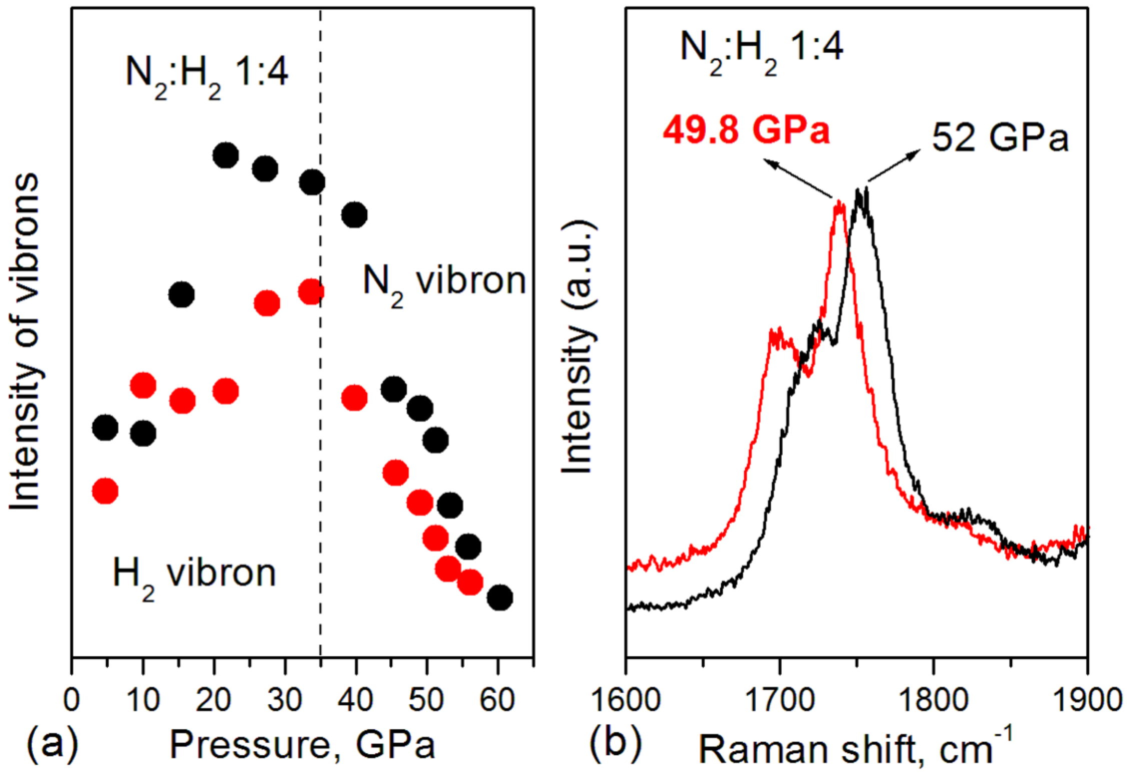

Fig. S3. Vibron changes at the polymerization. (a) The intensities of the hydrogen and nitrogen vibron substantially decrease at pressures above 35 GPa. (b) Raman spectra of ruby dependence of the time. Red line is recorded one hour later after compressing to 52 GPa. The auto-drop of pressure indicates a volume of the sample decrease during polymerization.

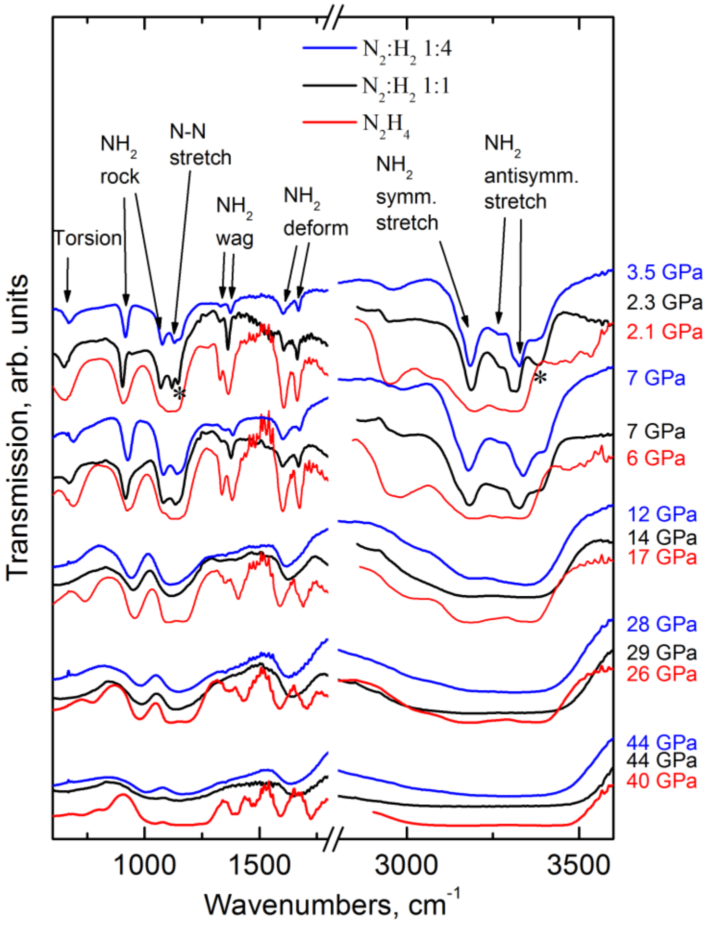

Fig. S4. IR spectra of the 1:4 (blue lines) and 1:1 (black lines) N_2:H_2 mixture compared with pure hydrazine (red lines). At a releasing pressure of 55 GPa after polymerization at 300 K. The spectra of the product did not change significantly with pressure down to 12 GPa. Below this pressure the spectra become very close to hydrazine. The only exceptions are lines marked with asterisks – they belong to ammonia. Ammonia appears in some runs, apparently the metallic gasket surrounding the sample catalyses this synthesis. Hydrazine absorption bands are assigned according to Ref. 19 of the main text.

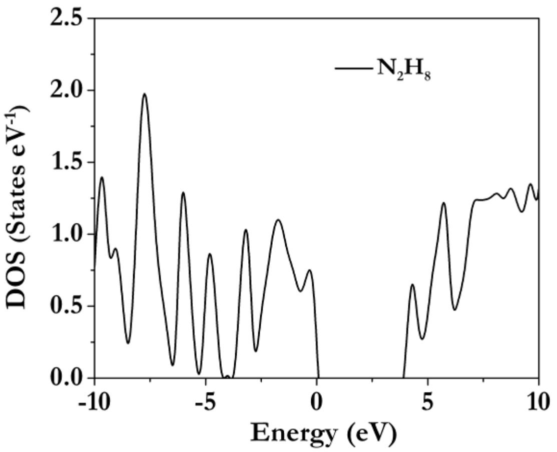

Fig. S5. The calculated total electronic density of states for N-H compound at 60 GPa is shown. The Fermi energy is set at zero. The band gap is ~3.7 eV.

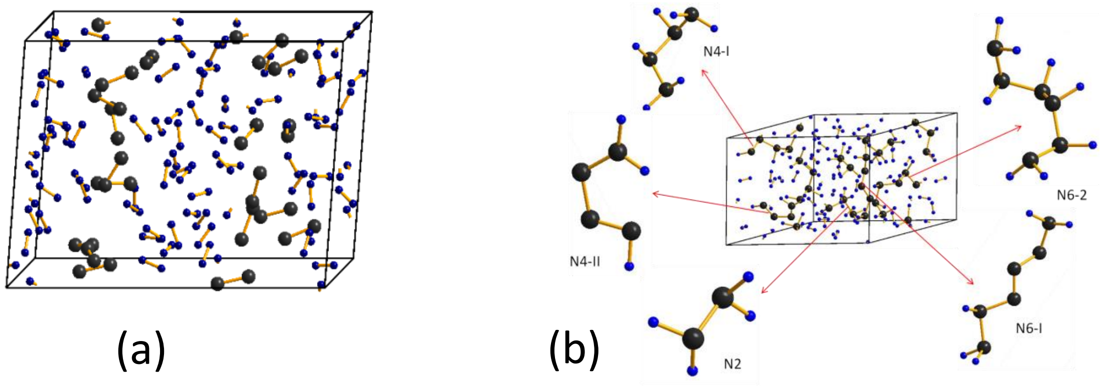

Fig. S6. Metadynamics theoretical prediction of high pressure structures. Predicted structure at (a) 30 GPa and 300 K and (b) 60 GPa at 500 K. The ratio of the polymeric units N2, N4I, N4II, N6I, N6II is: 4:3:1:1:1, correspondently.

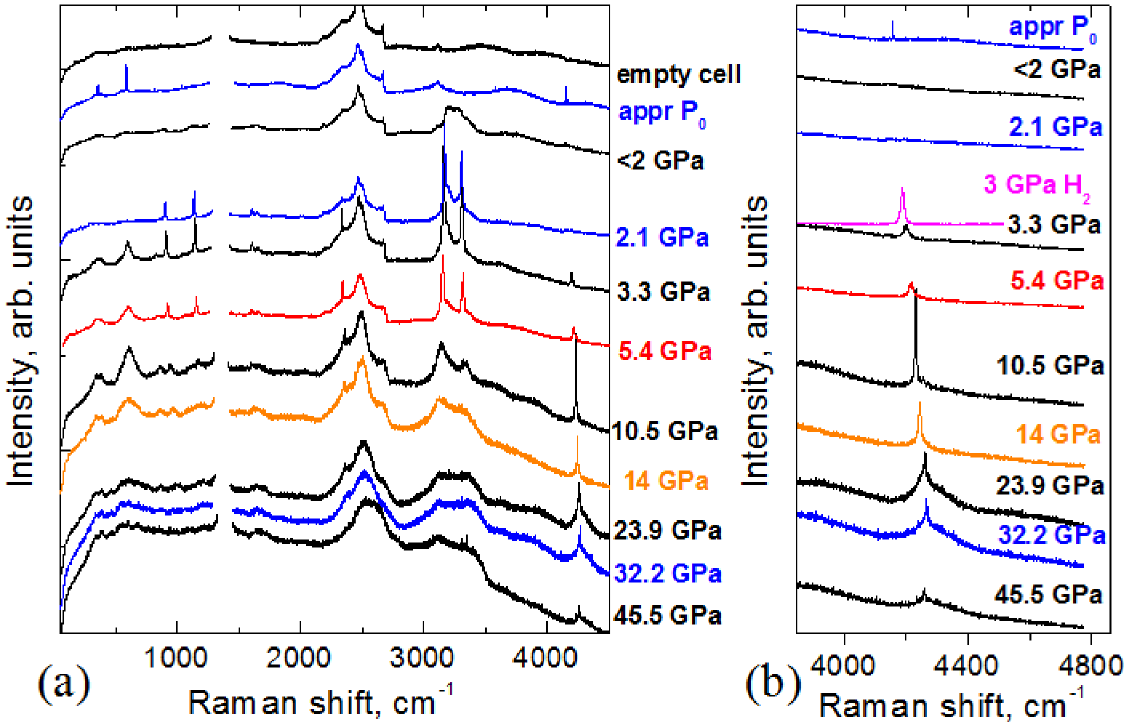

Fig. S7. Raman spectra of the 1:4 N_2:H_2 mixture at the releasing pressure. Evolution of the spectra (a) are similar to the run shown in Fig. 3a. Detailed changes of Raman spectra of hydrogen are shown in (b). At a releasing pressure below 10 GPa, hydrazine appears. Hydrogen remains inside hydrazine as its vibron is much broader than that for free hydrogen (magenta line). At the lowest pressures (not controlled) before opening the cell, hydrazine decomposes, and free hydrogen is released. Note, that molecular hydrogen presents in significant quantities in the oligomer as follows from a band at ~4300 cm^{-1} in the Raman spectra. A large broadening of this peak indicates that H_2 molecules interact with neighboring disordered oligomers. This situation is in contrast with the nitrogen crystal (Fig. S2b) where H_2 molecules reveal sharp pronounced vibrons.

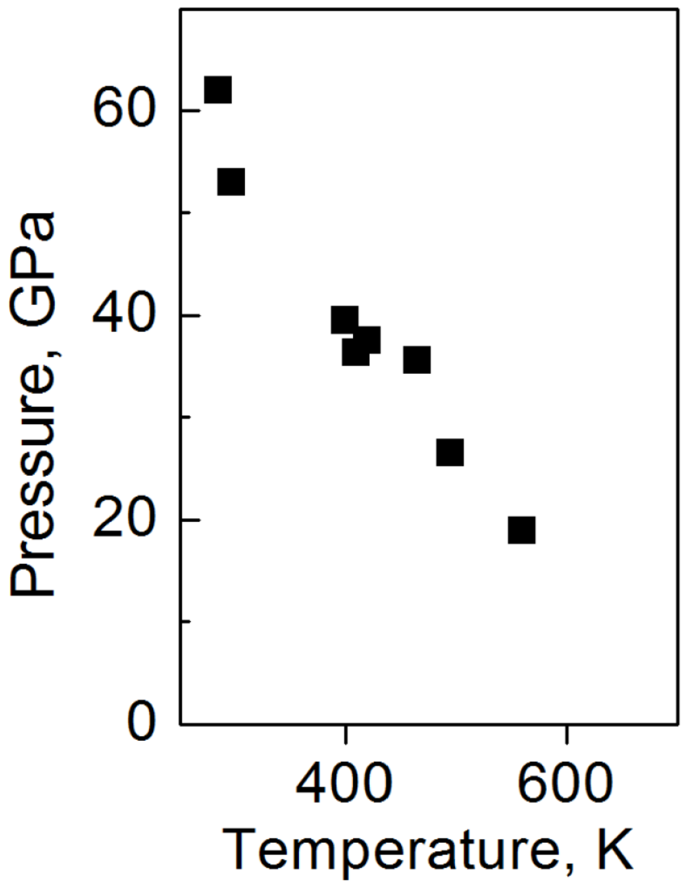

Fig. S8. The pressure-temperature conditions for the chemical transformation of the 1:1 N_2:H_2 mixture. Note that below room temperature the pressure needed for transformation significantly increases. At even colder temperatures of 200 and 250 K, we observed no chemical transformation even at pressures as high as 65 GPa. Higher temperatures substantially decrease the pressure of transformation.

Theoretical calculations on NH_2-$(NH)_n$-NH_2

A. Quantum chemical methodology

Density functional calculations were performed to explore the potential energy surfaces of hydrazine and NH_2-$(NH)_n$-NH_2 oligomers (azanes; azoalkanes) up to $N_{10}H_{12}$, determining geometries, relative energies, and vibrational frequencies. The reasons for choosing these oligomers is discussed in the section on IR vibrational spectra. The quantum chemical calculations were performed at the DFT level of theory, using the M06-2X functional (*1*) in conjunction with the aug-cc-pVDZ basis set. This level of theory is expected to be sufficiently accurate to analyze the trends in properties across the chain length. Some of the vibrational analysis are expected to be affected by complex features of the potential energy surface, e.g. for hydrazine (*2*–*5*). We do not aim to accurately reproduce these subtleties, which can induce errors in the fundamental frequencies of tens of cm^{-1}, but rather to examine how the vibrational modes vary with respect to chain length within the same level of approximation. Sample higher level calculations for hydrazine were conducted but the differences were found to be not significant for our current purpose.

The conformer space for smaller hydronitrogen chains, up to 6 nitrogen atoms in the backbone, was sampled exhaustively to determine the lowest energy conformers. The geometric patterns in these conformers were then generalized to longer chains up to 10 N-atoms, to obtain a good estimate of their optimal geometries. While this approach should give fairly good results, more stable conformers might still exist for these longer oligomers. For the current set of exploratory calculations only unbranched azane chains

were examined, with the exception of a cyclic structure. Also, only isolated molecules at zero pressure are examined theoretically at this time. As discussed below, this will impact some parts of the vibrational spectra.

All calculations were performed using the Gaussian-09 program suite (*6*). The ball-and-stick figures were prepared with Jmol.(*7*)

B. Computational results

B.1. Geometries

For hydrocarbon chains, the most stable conform is known to be the linear backbone, with all carbons in a plane. In contrast, the NH_2-$(NH)_n$-NH_2 chains show a preference for an N-N-N-N dihedral angle of about 76 degrees, causing the backbone to adopt a spiral shape (see Figure 4 in the main text); the left- and right-handed spirals were found to be enantiomers with identical properties. The most stable conformers also have all -NH- hydrogen atoms with H-N(N)N dihedral angles of the same sign. This spiral shape is clearly induced by the differences in interaction between the N-H bonds versus the lone pair on nitrogen, which is also the driving force in the hydrazine geometry. The inner N-N and N-H bond lengths of about 1.41 Å and 1.02 Å, respectively, are near-identical to those at the terminating N-NH_2 moiety (1.42 Å and 1.01 Å).

For cyclic N_nH_n compounds, no extensive conformer analysis was performed. For N_6H_6, a chair form was found to be the most stable structure, in agreement with earlier work (*8*, *9*). We refer to these latter publications, and the references therein, for an overview of the limited available work on cyclic azanes.

B.2. Thermodynamic stability

All NH_2-$(NH)_n$-NH_2 molecules were found to be comparatively stable energetic energy minima. Compared to the constituting elements in their elemental form, N_2 and H_2, the enthalpy of formation raises by about 20 kcal mol^{-1} for each added -NH- monomer (see *Table S1*). The successive endothermicities in elongating the chain are therefore near-identical as those for forming hydrazine. The formation enthalpy for the energetically most stable conformer, in all cases showing the spiral backbone discussed above, shows a slight dependence on the chain length, with a maximum at N_5H_7, and a modest decrease in formation enthalpy at 0 K after that (see *Table S1*). While this might suggest that, once past a certain chain length, elongating an existing chain further might be energetically more favorable than forming shorter chains, the energy differences are too small to be significant at the current level of theory. The peak in enthalpy increase around N_5H_7 is related to the length of the spiral, where 5 to 6 nitrogen atoms is the minimum length to form a full revolution of the spiral backbone.

B.3. Formation and decomposition pathways

The chemical pathways in forming, elongating, and breaking the chains seems to involve a complex set of radical reactions. Their study is well beyond the scope of this work, and the reader is referred to the literature on the analogous hydrazine (catalytic) combustion for possible reaction candidates. Unimolecular H-elimination is easier for the inner hydrogen, with a bond strength of 68 kcal mol^{-1} compared to 74 for the -NH_2 hydrogens. H_2-elimination at the terminal -NH_2 group requires 69 kcal mol^{-1}. Breaking of the nitrogen chain requires 50 kcal mol^{-1} at the chain end, forming $\cdot NH_2$ radicals, but only

30 kcal mol^{-1} for the inner N-N bonds, forming a pair of -N$^{\bullet}$H + $^{\bullet}$NH- radicals. This latter pathway is significantly below the unimolecular decomposition channels for hydrazine and its alkylated forms, the lowest of which involves breaking of the N-N bond with a barrier of at least 60 kcal mol^{-1}.

Table S1: Formation enthalpy (kcal mol^{-1}) at zero Kelvin, and relative energies (kcal mol^{-1}) upon elongating the chain by one NH monomer.

**Compound**	**ΔH(0K)**	**E_{rel}(N_xH_x to $N_{x-1}H_{x-1}$)**
N_2+H_2	0.0	
N_2H_4	19.1	19.1
N_3H_5	39.0	19.9
N_4H_6	58.8	19.8
N_5H_7	79.43	20.6
N_6H_8	109.1	19.9
N_7H_9 [a]	119.2	19.8
N_8H_{10} [a]	139.0	19.8
N_9H_{11} [a]	158.6	19.7
$N_{10}H_{12}$ [a]	178.2	19.6

B.4. Infrared vibrational spectrum

The gas phase vibrational spectra obtained theoretically are only partially comparable to those in liquid and solid phase. In particular, vibrational modes involving large skeletal movements are more hindered in the solid phase, often with significant shifts in the vibrational frequencies. Still, we can compare several spectral bands between hydrazine and the oligomers, and use the results to qualitatively support the experimentally observed spectral changes. The experimental data is expected to be obtained for a mixture of many nitrogen-hydrogen compounds ranging, of unknown chain length, branching, cyclic forms, or saturation. Comparison of the experimental IR spectra to the theoretically predicted fundamental wavenumbers for individual compound conformers is therefore not overly revealing. Below, we instead show spectra of mixtures of compounds, so the dependence of the spectrum on the mixture can be visualized. Two elementary spectra, for hydrazine (Fig. S9) and cyclic N_6H_6 (Fig. S10) are shown, as well as the spectra for mixtures containing a) the lowest conformer characterized for each of azanes from N_3 to N_{10} in equal concentrations (Fig. S11), b) all conformers of N_3H_5 in equal proportion (Fig. S12), c) all conformers of N_4H_6 in equal proportion (Fig. S13), d) all conformers of N_5H_7 in equal proportion (Fig. S14), e) all conformers of N_6H_8 in equal proportion (Fig. S15) and f) all conformers for N_3H_5 through N_6H_8 with each conformer in equal concentration (Fig. S16). These synthetic spectra were generated from the vibrational modes and their IR intensities as predicted from the M06-2X/aug-cc-pVDZ calculations, assuming gaussian-function-shaped peaks with an arbitrary width at half height of about 8 cm^{-1}, and renormalized to the strongest absorption peak.

An important difference between the cyc-N_6H_6 spectrum, compared to the experimental spectrum and the spectra for the linear azanes is the absence of peaks near 1650 cm^{-1}, which corresponds to the NH_2 wagging modes. The prominence of this peak in the experimental spectrum suggests strongly that unsubstituted cyclic azanes can therefore not be the main products. As such, we exclude cyclic oligomers from our discussion below.

The NH stretch vibrations all have wavelengths above 3400 cm^{-1}, both for the oligomers and hydrazine. In hydrazine, the coupling between the eigenstates of the C-H vibrations of the -NH_2 moiety leads to their separating into a symmetric and an antisymmetric stretch. For the oligomers, the bulk of the hydrogen atoms are singletons, leading to much weaker coupling and no clear splitting of the spectrum, as observed experimentally. Furthermore, the reaction vessel is expected to contain a mixture of different oligomers lengths, branching, and degree of folding, each affecting the surroundings of the individual hydrogen atoms slightly, and hence leading to slightly different vibrational frequencies. For the longer oligomers, N_9H_{11} and $N_{10}H_{12}$, we already predict vibrational modes ranging from 3424 to 3613 cm^{-1} with the individual frequencies spread out more or less evenly across this band. This seems to support the broad peak around 3500 cm^{-1} seen in the experiment. Oligomer branching should introduce further peak broadening, as this introduces further variations of the H-atom environments.

A second spectral band of interest are the peaks just above 1600 cm^{-1}. In hydrazine, these correspond to the wagging of the two NH_2 moieties; these modes again couple strongly into a symmetric and antisymmetric eigenfunction resulting in a split-peak spectrum. For N_3H_5, the predicted vibrational NH_2 wagging modes are already significantly less

coupled, with each mode showing a large-amplitude wagging on one NH_2 end, and only a small amplitude wagging at the other NH_2 moiety. Upon further elongation of the nitrogen backbone, the two NH_2 wagging modes become fully localized on one or the other -NH_2 moiety. This decoupling of the NH_2 wagging mode, combined with small changes in the NH_2 environment by chain branching and folding, would prevent the formation of two separate peaks, but rather yield a single peak, as is observed experimentally. Again, oligomer branching should broaden this peak further.

For the vibrational spectrum of the oligomers at frequencies below 1500 cm^{-1}, the gas phase predictions are not expected to be accurate, as larger amplitude skeleton vibrations behave differently in solid phase, with typically a shift towards higher frequencies. This is particularly true for the broad peak in the spectra below 700 cm^{-1}. Rather than over-interpreting the predicted oligomer spectrum in this region, we merely note that the experimental spectra and the theoretically predicted spectra remain well comparable. A more accurate comparison of the spectra requires the application of different computational techniques explicitly calculating spectra for the high-pressure solid phase.

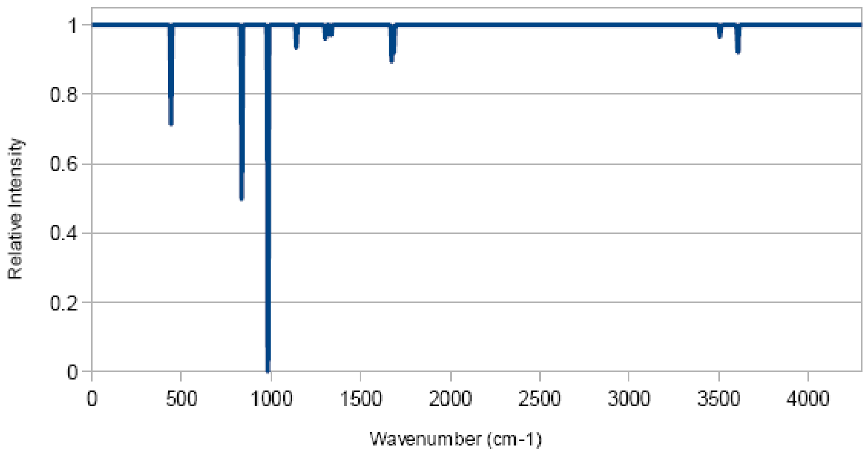

Fig. S9: Absorption spectrum of hydrazine as obtained at the M06-2X/aug-cc-pVDZ level of theory. Simulated with a peak width at half height of 8 cm^{-1}.

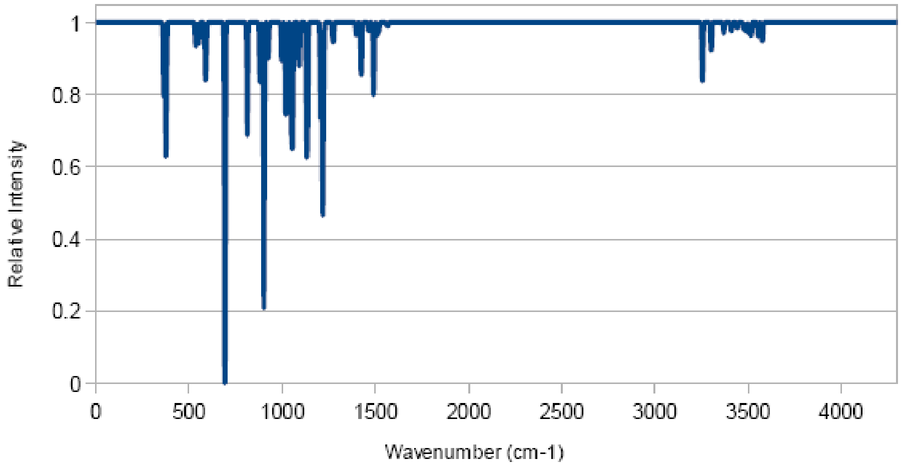

Fig. S10: Absorption spectrum of cyclic-N_6H_6 as obtained at the M06-2X/aug-cc-pVDZ level of theory. Simulated with a peak width at half height of 8 cm^{-1}.

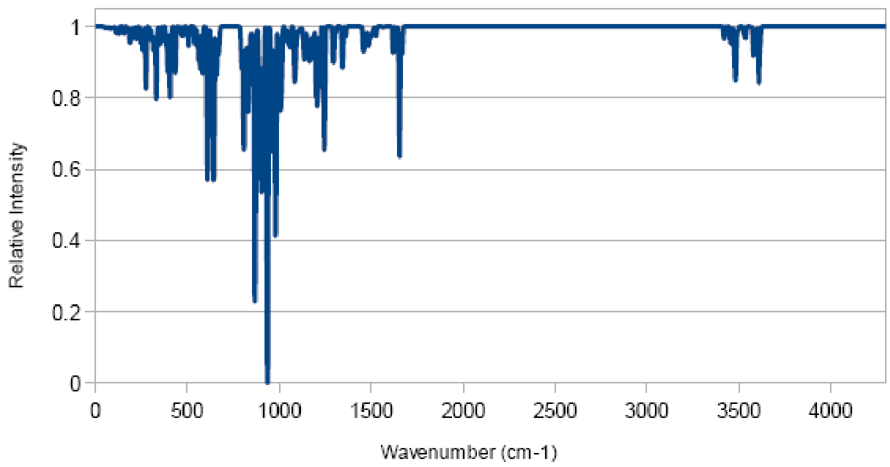

Fig. S11. Absorption spectrum of a mixture of N_3H_5 through $N_{10}H_{12}$, with one conformer each in equal concentration, as obtained at the M06-2X/aug-cc-pVDZ level of theory. Simulated with a peak width at half height of 8 cm^{-1}.

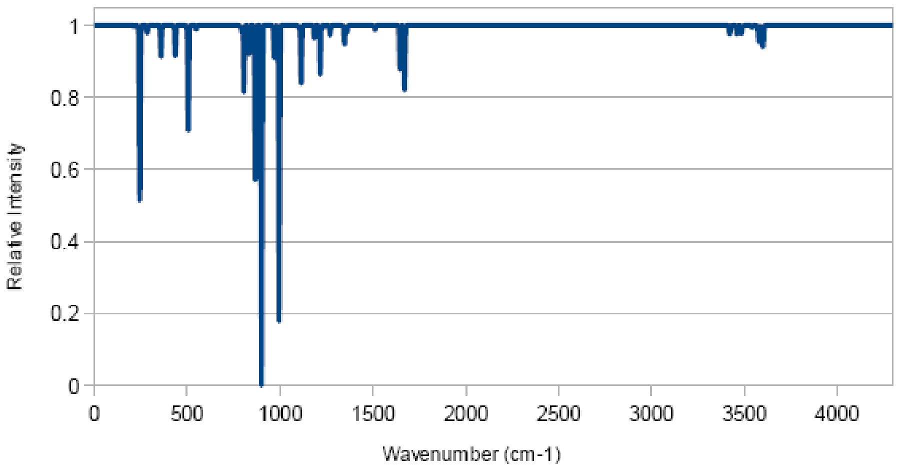

Fig. S12: Absorption spectrum of a mixture of N_3H_5 conformers in equal concentration, as obtained at the M06-2X/aug-cc-pVDZ level of theory. Simulated with a peak width at half height of 8 cm^{-1}.

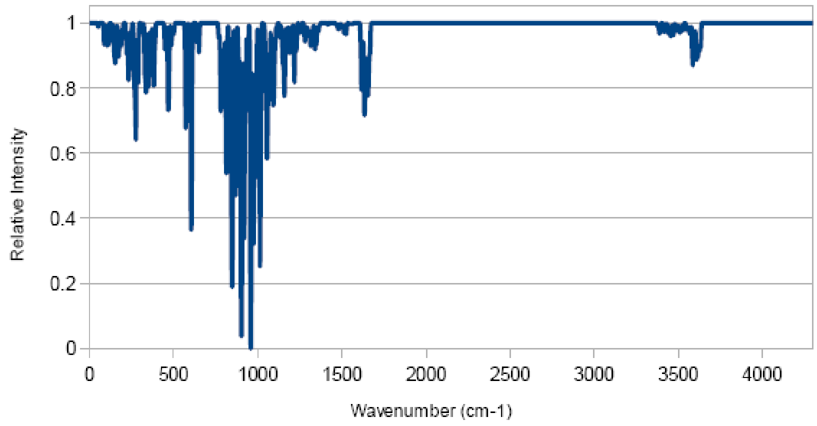

Fig. S13: Absorption spectrum of a mixture of N_4H_6 conformers in equal concentration, as obtained at the M06-2X/aug-cc-pVDZ level of theory. Simulated with a peak width at half height of 8 cm^{-1}.

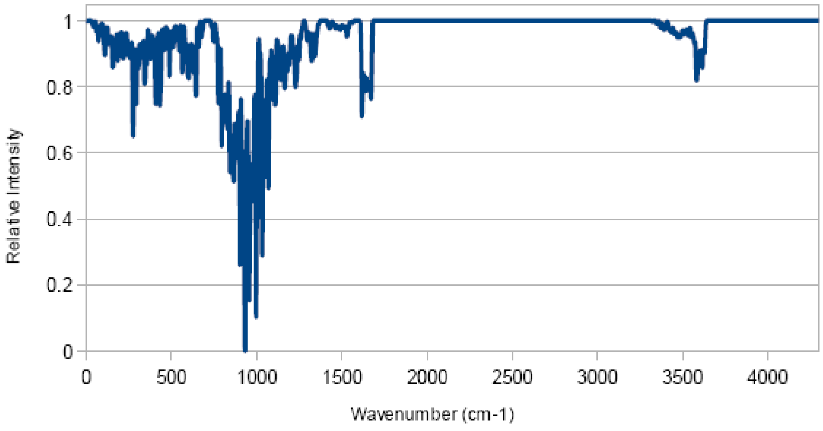

Fig. S14: Absorption spectrum of a mixture of N_5H_7 conformers in equal concentration, as obtained at the M06-2X/aug-cc-pVDZ level of theory. Simulated with a peak width at half height of 8 cm^{-1}.

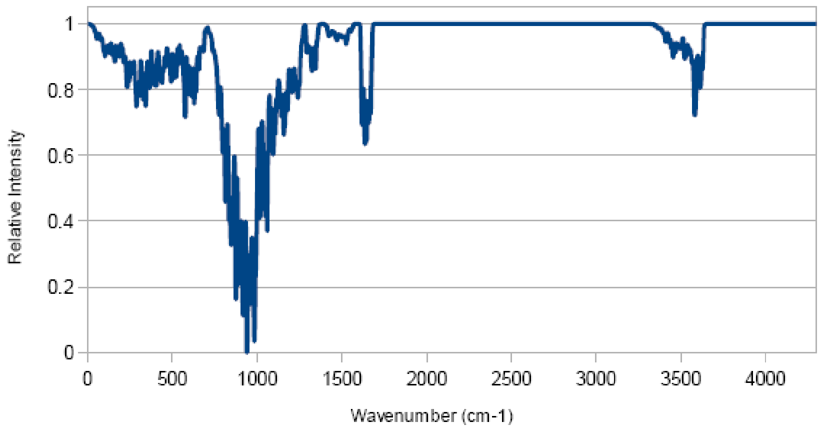

Fig. S15: Absorption spectrum of a mixture of N_6H_8 conformers in equal concentration, as obtained at the M06-2X/aug-cc-pVDZ level of theory. Simulated with a peak width at half height of 8 cm^{-1}.

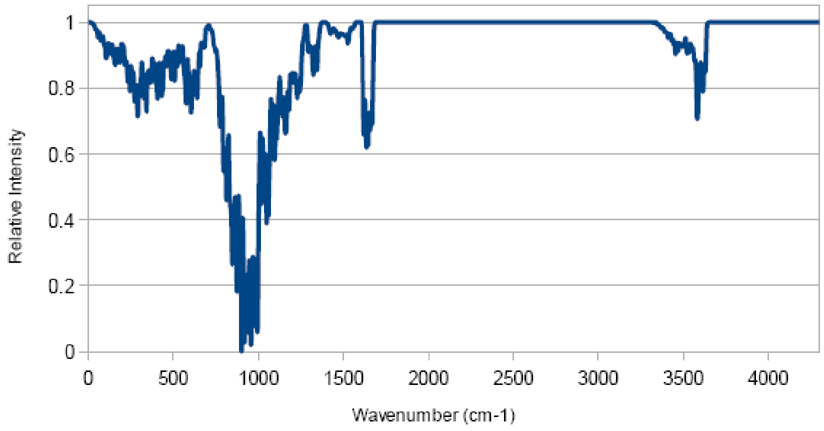

Fig. S16: Absorption spectrum of a mixture of all N_3H_5 through N_6H_8 conformers in equal concentration, as obtained at the M06-2X/aug-cc-pVDZ level of theory. Simulated with a peak width at half height of 8 cm^{-1}.

References

1. Y. Zhao, D. G. Truhlar (2008) *Theor. Chem. Accounts* **120**, 215–241

2. D. W. Ball (2001) *J. Phys. Chem. A* **105**, 465–470

3. F. B. C. Machado, O. Roberto-Neto (2002) *Chem. Phys. Lett.* **352**, 120–126

4. A. T. Kowal (2003) *J. Mol. Struct. Theochem* **625**, 71–79

5. W. Łodyga, J. Makarewicz (2012) *J. Chem. Phys.* **136**, 174301.

6. M. J. Frisch *et al.*, *Gaussian 09, Revision B.01* (Gaussian Inc., Wallington CT, 2010).

7. *Jmol: an open-source Java viewer for chemical structures in 3D* (www.jmol.org).

8. H.-Y. Wu, W.-F. Cai, L.-C. Li, A.-M. Tian, N.-B. Wong (2011) *J. Comput. Chem.* **32**, 2555–2563

9. B.-H. Xu, L.-C. Li, L. Sun, A.-M. Tian (2008) *J. Mol. Struct. Theochem* **870**, 77–82
